# Hepato(Geno)Toxicity Assessment of Nanoparticles in a HepG2 Liver Spheroid Model

**DOI:** 10.3390/nano10030545

**Published:** 2020-03-18

**Authors:** Elisabeth Elje, Espen Mariussen, Oscar H. Moriones, Neus G. Bastús, Victor Puntes, Yvonne Kohl, Maria Dusinska, Elise Rundén-Pran

**Affiliations:** 1 Health Effects Laboratory, Department for Environmental Chemistry, NILU—Norwegian Institute for Air Research, Instituttveien 18, 2007 Kjeller, Norway; eel@nilu.no (E.E.); ema@nilu.no (E.M.); mdu@nilu.no (M.D.); 2 Department of Molecular Medicine, Institute of Basic Medical Sciences, Faculty of Medicine, University of Oslo, Sognsvannsveien 9, 0372 Oslo, Norway; 3 Institut Català de Nanociència y Nanotecnologia (ICN2-UAB-CSIC-BIST), Campus UAB, Bellaterra, 08193 Barcelona, Spain; oscarhernando.moriones@icn2.cat (O.H.M.); victor.puntes@icn2.cat (V.P.); 4 Universitat Autonòma de Barcelona (UAB), Campus UAB, Bellaterra, 08193 Barcelona, Spain; 5 Vall d’Hebron Institut de Recerca (VHIR), 08035 Barcelona, Spain; 6 Institució Catalana de Recerca i Estudis Avançats (ICREA), 08010 Barcelona, Spain; 7 Fraunhofer Institute for Biomedical Engineering IBMT, Joseph-von-Fraunhofer-Weg 1, 66280 Sulzbach, Germany; yvonne.kohl@ibmt.fraunhofer.de

**Keywords:** advanced in vitro model, comet assay, genotoxicity, hepatotoxicity, liver spheroids, nanoparticles, 3D culture, HepG2

## Abstract

(1) In compliance with the 3Rs policy to reduce, refine and replace animal experiments, the development of advanced in vitro models is needed for nanotoxicity assessment. Cells cultivated in 3D resemble organ structures better than 2D cultures. This study aims to compare cytotoxic and genotoxic responses induced by titanium dioxide (TiO_2_), silver (Ag) and zinc oxide (ZnO) nanoparticles (NPs) in 2D monolayer and 3D spheroid cultures of HepG2 human liver cells. (2) NPs were characterized by electron microscopy, dynamic light scattering, laser Doppler anemometry, UV-vis spectroscopy and mass spectrometry. Cytotoxicity was investigated by the alamarBlue assay and confocal microscopy in HepG2 monolayer and spheroid cultures after 24 h of NP exposure. DNA damage (strand breaks and oxidized base lesions) was measured by the comet assay. (3) Ag-NPs were aggregated at 24 h, and a substantial part of the ZnO-NPs was dissolved in culture medium. Ag-NPs induced stronger cytotoxicity in 2D cultures (EC_50_ 3.8 µg/cm^2^) than in 3D cultures (EC_50_ > 30 µg/cm^2^), and ZnO-NPs induced cytotoxicity to a similar extent in both models (EC_50_ 10.1–16.2 µg/cm^2^). Ag- and ZnO-NPs showed a concentration-dependent genotoxic effect, but the effect was not statistically significant. TiO_2_-NPs showed no toxicity (EC_50_ > 75 µg/cm^2^). (4) This study shows that the HepG2 spheroid model is a promising advanced in vitro model for toxicity assessment of NPs.

## 1. Introduction

During the last decades, concerns have been raised about the potential human health risk of nanoparticles (NPs) due to the increased development and production of NPs with novel properties [1,2]. NPs are produced in a huge variety of forms and in large volumes, and they are used in a broad range of applications in everyday life. For example, NPs of titanium dioxide (TiO_2_) are used as a pigment in paint, food and cosmetics [3]; zinc oxide (ZnO) is used in cosmetics due to its UV-blocking properties [4]; and silver (Ag) is used as a disinfection agent in medical equipment and consumer products, on account of its antimicrobial activity [5]. Thus, humans are likely to be exposed to NPs, either intentionally or accidentally, during production and usage [6]. Transport of NPs across biological barriers has been observed by elemental analysis in both rodents and humans [7,8,9,10,11]. As an example, gold NPs have been reported to reach the systemic circulation in humans, after inhalation, and translocate to other organs [8,9].

Several *in vivo* studies show that NPs accumulate in the liver, which is an important target organ for NPs and other xenobiotics due to its metabolic activity [12,13,14,15,16,17,18]. Induction of hepatotoxicity is one of the most common reasons for a medicine to be rejected or removed from the market [19,20]. Therefore, there is a need for sensitive hepatotoxicity screening methods for drug development and hazard assessment of chemicals or new materials, such as NPs. When considering the 3Rs—replacement, reduction and refinement—to minimize the use of animal experiments, hepatotoxicity should be assessed by reliable in vitro models. A great advantage of in vitro hepatocellular models for studying hepatotoxicity is the possibility of using human cells, either as primary cells or cell lines. The use of human hepatocyte cell lines, such as HepG2, C3A, Huh7 and HepaRG, has many advantages compared to primary cells. They are relatively easy to culture and have an unlimited life span, a relatively stable phenotype, high availability and low costs; moreover, inter-donor variations are avoided [21]. However, when comparing in vitro cell culture models in standard two-dimensional (2D) monolayers with complex organs, the cell lines in 2D culture display a limited hepatocytic functionality [21].

The liver-like functionality of the human hepatocellular carcinoma cell line HepG2 is enhanced when the cells are cultured in a three-dimensional (3D) arrangement. This increases the cell-to-cell contacts and intercellular communication [22] and changes the protein expression and metabolic status of the cells [21,22,23]. HepG2 cells in 3D cultures show upregulation of genes involved in liver-specific xenobiotic and lipid metabolism, whereas genes related to the extracellular matrix, cytoskeleton and cell adhesion have higher expression in 2D cultures [22,24].

The use of spheroids as 3D cultures in hepatotoxicity assessment is an increasing field of interest, and HepG2 spheroids, prepared with and without using scaffolds, have been applied for toxicity experiments with both NPs [6,25,26] and chemicals [27,28]. However, the differences in toxic responses between cells cultured in 2D and 3D are not yet clear. The scaffold-free HepG2 spheroid model was characterized in [27], where we demonstrated its applicability for testing genotoxicity of standard chemicals by the modified enzyme-linked comet assay, which measures DNA strand breaks (SBs) and oxidized DNA lesions. Interestingly, we found differences in sensitivity between the 2D and 3D models [27]. The comet assay has also been performed with Ag-, ZnO- and TiO_2_-NPs and carbon nanotubes on a commercialized spheroid model with primary liver cells [6], and it has been shown to work well with different 3D models [6,27,29,30]. However, the comet assay has—to our knowledge—not yet been applied in HepG2 spheroids for genotoxicity testing of NPs. By using the miniaturized version of the comet assay, the throughput is increased. High-throughput methods are needed to reduce and replace animal experiments and to align with the increasing amounts of NPs being produced [31]. HepG2 spheroids have also been applied in the micronucleus test for chromosomal aberration testing, showing higher sensitivity than a standard 2D model to exposure to benzo(a)pyrene and 2-amino-1-methyl-6-phenylimidazo(4,5-b)pyridine [28]. In contrast, Dubiak-Szepietowska et al. (2016) found that liver 3D cultures are more resistant than 2D to cytotoxicity induced by NPs of Ag, SiO_2_ and ZnO [25].

This study aimed to evaluate cytotoxicity and genotoxicity in HepG2 2D and 3D cultures after 24 h exposure to TiO_2_-, Ag- and ZnO-NPs and to identify any differences in responses in 2D and 3D cultures. The tested NPs were selected on the basis of high production volumes and applications in consumer and medical products.

## 2. Materials and Methods

### 2.1. Cultivation of HepG2 Cells and Preparation of Spheroidal Cultures

HepG2 cells, provided from the ECACC (European Collection of Authenticated Cell Cultures) (cell line no. 85011430, Salisbury, United Kingdom) were cultured in 2D and 3D arrangements, as previously explained in detail [27]. In brief, HepG2 cells were cultured in Dulbecco’s modified Eagle’s medium (DMEM D6046 with low glucose and 4 mM L-glutamine, Sigma-Aldrich, Oslo, Norway) supplemented with 10% *v*/*v* fetal bovine serum (FBS, 26140-079, Thermo Fisher Scientific, Oslo, Norway), 100 U/mL penicillin and 100 µg/mL streptomycin (5070-63, Thermo Fisher Scientific, Oslo, Norway). Spheroid generation was performed, using the hanging drop technique, with 2500 cells per 20 µL drop. After four days of incubation of the cells at 37 °C with 5% CO_2_ as hanging drop, the spheroids were transferred to a low adhesion plate. After one week, the spheroids had a diameter of approximately 800 µm [27] and were exposed to NPs as explained below. In parallel, 2D cultures were seeded in a 96-well plate with 20000 cells/well the day before exposure.

### 2.2. Nanoparticle Dispersions and Preparation for Toxicity Studies

TiO_2_-NPs were provided by Catalan Institute of Nanoscience and Nanotechnology (ICN2, Spain) in colloidal dispersion and stored at 4 °C. TiO_2_-NPs, of a mean size of approximately 4 nm in diameter, were prepared by a precipitation method following and adapting the method of Pottier et al. [32]. The stock solution of Ti^4+^ (0.7 M) was prepared by dissolving the Titanium (IV) isopropoxide (TTIP, Fluka Chemika) precursor in an HCl (3 mol/L) solution. For the production of TiO_2_ anatase NPs, an aqueous Ti^4+^ stock solution (50 mL) was diluted in Milli-Q water (350 mL), at room temperature. The pH of the mixture was fixed at 11 by the addition of NaOH (3 M). Suspensions were aged at 70 °C for 24 h, and the solid was collected by centrifugation. Samples were further purified by 3 centrifuging cycles and re-suspended in an aqueous solution of tetramethylammonium hydroxide (TMAOH, Sigma-Aldrich) (100 M). Samples were characterized by transmission electron microscopy, dynamic light scattering and UV-Vis spectroscopy. The former was used to determine the particle size and size distribution. On the day of exposure, the TiO_2_-NPs were diluted in FBS (1:1), and thereafter diluted 1:9 in cell culture medium, without FBS, to a concentration of 455 µg/mL (stock dispersion).

Ag-NPs (NM300K) were provided by Fraunhofer Institute for Molecular Biology and Applied Ecology (IME, Schmallenberg, Germany) and ZnO-NPs (NM110, JRCNM01100a) by the Joint Research Centre (Ispra, Italy). Stock dispersions of Ag- and ZnO-NPs were prepared according to the NANOGENOTOX protocol [33]. Briefly, the Ag- or ZnO-NPs were mixed with a bovine serum albumin (BSA) water solution (0.05% *m*/*v*, product nr A9418, Sigma-Aldrich), in a 20 mL scintillation vial (Wheaton Industries, Millville, NJ, USA), to a final concentration of 2.56 mg/mL (stock dispersion). To the ZnO-powder, 30 µL 100% ethanol (product nr 600068, Antibac AS, Asker, Norway) per 15.4 mg NP powder was added before BSA-water, to facilitate dispersion. The NP/BSA–water mixtures were sonicated on ice with a sonicator probe Labsonic^®^P Probe 3 mm (853 5124, Sartorius Stedim Biotech, Göttingen, Germany) and Labsonic^®^P (Sartorius Stedim Biotech), at 50% amplitude for 15 min (100% cycle, 100 watts), or with an ultrasound homogenizer Sonopuls (Bandelin, Germany), at 50% amplitude for 15 min (100% cycle). The dispersion solution of NM300K (NM300K DISP) was not included in the study, based on negative results from other studies on cytotoxicity and DNA damage [34,35,36].

Working concentrations of all the NPs were prepared by serial dilution of the stock dispersion in the culture medium. HepG2 cells in 2D and 3D culture were exposed for 24 h to TiO_2_-NPs, Ag-NPs or ZnO-NPs (1–75 µg/cm^2^ in 2D system, corresponding to 3–212 µg/mL in both systems; see Supplementary Tables S1 and S2 for details). As a negative control, complete NP-free culture medium was used. The same volumes (100 µL per well) and concentrations of NPs were used for both 2D and 3D cultures. NP dispersions were prepared, at most, two hours before cell exposure and NP characterization.

For characterization purposes (next sections), the NP stock solutions were also prepared in water dispersions, without proteins present, as described above, but in water instead of FBS/medium and BSA-water.

### 2.3. Size and Morphology Measurements of the NPs by Electron Microscopy

TiO_2_-NP diameters were obtained from the analysis of transmission electron microscopy (TEM) images acquired with a FEI Tecnai G2 F20 S-TWIN HR(S) TEM equipped with an energy-dispersive X-ray spectroscopy (EDX) detector, operated at an accelerated voltage of 200 kV. Microliters of the samples were prepared by drop-casting 10 μL of the sample on a carbon-coated copper TEM grid and leaving to dry at room temperature. In addition, scanning electron microscopy was done with a FEI Magellan 400L XHR SEM, in scanning mode, operated at 1 kV, and in transmission mode, operated at 20 kV/STEM, for bigger sizes. The average size and size distribution of the samples were measured by using ImageJ software, by counting at least 300 particles from different regions of the grid. TEM images of Ag- and ZnO-NPs were acquired, but the size distribution was not measured.

### 2.4. Hydrodynamic Diameter and Zeta Potential Measurements of the NPs

The hydrodynamic size and surface charge of the NPs were determined by Dynamic Light Scattering and Laser Doppler Anemometry, respectively, using a Zetasizer Nano ZS (Malvern Instruments, Malvern, UK) instrument equipped with a light source wavelength of 532 nm and a fixed scattering angle of 173°. Aliquots of one milliliter of the colloidal NP dispersions at a concentration of 10% (*v*/*v*) were placed into specific plastic cuvettes, and the software was arranged with the parameters of refractive index and absorption coefficient, and the solvent viscosity at 25 °C. Each value was the average of at least 3 independent measurements. All measurements used the Smoluchowski model.

### 2.5. UV-Vis Measurements of the NP Dispersions

UV-visible spectra were acquired with an Agilent Cary 60 UV-Vis spectrophotometer. A 10% (*v*/*v*) colloidal NP dispersion was placed in a cell, and the spectral analysis was performed in the 200–800 nm wavelength range, at room temperature.

### 2.6. Analysis of Silver and Zinc Ions in NP Dispersions

Samples for analysis of dissolved silver and zinc and potential dissolution of the NPs were taken from cell-free exposure medium parallel to the start of exposure. The concentrations 1, 10, 30 and 100 µg/cm^2^ were selected, corresponding to 2.8–283 µg/mL. Medium without NPs was used as control. The samples were transferred to Amicon Ultra centrifugal filter unit tubes (Millipore, product no UFC900324) containing a 3 KDa filter unit [37]. The tubes were preconditioned before use with ultrapure water at 3900 g for 10 min. The samples were centrifuged at 3900 g for 30 min, to let the particles remain in the filter and the dissolved Ag and Zn to go through with the filtrate.

An aliquot of the filtrate containing released ions from the NPs was added to supra pure nitric acid, at a final concentration of 1% (*v*/*v*). The concentrations of dissolved zinc and silver (defined as <3 kDa fraction) were determined by the use of an inductively coupled plasma mass spectrometer (ICP-MS) type Agilent 7700× (Agilent, Santa Clara, CA, USA), using the method accredited according to requirements of NS-EN/IEC 17025 (NILU-U-110). Then, ^115^In was added to all standards, blanks and samples, as internal standard, and detection limits were 0.006 ng/mL Ag and 0.6 ng/mL Zn. Certified reference material (1640a Trace Elements in Natural Water, NIST) were analyzed in every run. One sample per concentration was used in three independent experiments (*n* = 3).

### 2.7. Fluorescence Imaging of the Spheroids

After NP exposure, the spheroids were washed with PBS before live and dead cells were stained by fluorescein diacetate (FDA, Invitrogen, Thermo Fisher Scientific, Oslo, Norway) and propidium iodide (PI, Invitrogen, Thermo Fisher Scientific), respectively. After incubation with 30 µg/mL FDA and 40 µg/mL PI for 10 min in the dark, at room temperature, the spheroids were washed with PBS and transferred to a glass-bottomed culture slide (µ-slide 8-well glass bottom, Ibidi) for imaging with confocal microscope Zeiss LSM 700, using the software ZEN2010 (Zeiss). Excitation and emissions peaks were 535 and 617 nm for PI and 498 and 517 nm for FDA. At least three spheroids were imaged from each sample in two independent experiments (*n* = 2). Z-stack images were captured from the spheroid surface and approximately 150 µm inside, toward the center of the spheroid, as described in [27]. The images were merged by using maximum intensity in ImageJ [38].

### 2.8. Viability Measurements by AlamarBlue Assay

The alamarBlue assay measures the ability of the cells to metabolize resazurin by reducing it to the fluorescent molecule resorufin. The metabolic capacity represents the viability of the cell culture relative to the control sample. The assay was performed to evaluate the cell viability in the 3D and 2D cultures after NP exposure, as described in [27]. In brief, 2D and 3D cultures were washed with PBS and incubated with alamarBlue solution (10% *w*/*v*) for 3 h, before fluorescence was measured quantitatively on a plate reader (excitation = 530 nm; emission = 590 nm). Chlorpromazine hydrochloride (Sigma-Aldrich, Oslo, Norway), 100 µM, was included as positive control for the assay, based on results from [27], giving cell viability below 30% for both 2D and 3D cultures after 24 h exposure. At least two and three parallel culture wells were used per concentration for 2D and 3D cultures, respectively, and at least two wells per culture well were used for determining average fluorescence. To control for potential interference between the NPs and the alamarBlue solution, cell-free control samples, with and without NPs, were included.

To compare potential cytotoxic effects of NPs with their corresponding salts, HepG2 cells in 2D configuration were exposed to solutions of silver nitrate (AgNO_3_) and zinc chloride (ZnCl_2_). Both AgNO_3_ (product nr 319430, Fluka) and ZnCl_2_ (product nr 793523, Sigma-Aldrich, Oslo, Norway) were dissolved in complete cell culture medium (5 mM) before being further diluted upon cell exposure (1–5000 µM). Cells (2D) were exposed in 96-well plates, with at least 3 parallel exposure wells, for 24 h.

### 2.9. DNA Damage Measured by the Comet Assay

The enzyme-linked alkaline comet assay with inclusion of formamidopyrimidine DNA glycosylase (Fpg, gift from NorGenoTec AS Professor Andrew Collins and Dr. Sergey Shaposhnikov, Norway) was used to measure the level of DNA SBs and oxidized bases in 2D and 3D cultures. Fpg measures oxidized and ring open purines and DNA alkylated bases [39,40] and converts these lesions to SBs. The detailed procedure of the modified comet assay in 2D and 3D models is described in [27]. In brief, disaggregated cultures were embedded in low-melting-point agarose on precoated slides, before being submerged in lysis solution (2.5 M NaCl, 0.1 M EDTA, 10 mM Tris, 10% *v*/*v* Triton X-100, pH 10, 4 °C) for at least 1 h. The miniaturized version of the comet assay was used, with 12 mini-gels on each slide, similarly to [36]. Slides with samples for Fpg incubation were washed twice for 8 min in buffer F (40 mM HEPES, 0.1 M KCl, 0.5 mM EDTA, 0.2 mg/mL BSA, pH 8, 4 °C), Fpg diluted in buffer F was added, covered with a polyethylene foil and incubated at 37 °C for 30 min, in a humid box. All slides with cells embedded in gels were placed in the electrophoresis tank with electrophoresis solution (0.3 M NaOH, 1 mM EDTA, pH > 13, 4 °C), to let the DNA unwind for 20 min before running electrophoresis for 20 min (25 V, 1.25 V/cm, Consort EV202). Slides were neutralized in PBS and H_2_O and dried horizontally, before staining with SYBR^®^gold (Sigma-Aldrich, Oslo, Norway). Comets were imaged using a Leica DMI 6000 B microscope (Leica Microsystems), equipped with a SYBR^®^photographic filter (Thermo Fischer Scientific, Oslo, Norway), and scored using the software Comet Assay IV 4.3.1 (Perceptive Instruments, Bury St Edmunds, UK). Median % DNA in tail from around 50 comets per gel was used as a measure of DNA SBs. Oxidized DNA lesions were calculated as net Fpg-sensitive sites, i.e., as the difference in % DNA in tail between samples with Fpg incubation and samples without incubation. Hydrogen peroxide, H_2_O_2_ (50 µM, Sigma-Aldrich, Oslo, Norway), and the photosensitizer Ro 19-8022 (2 µM, kindly provided by Hoffmann La Roche) with light irradiation were included as positive controls for DNA SBs and Fpg activity, respectively. The photosensitizer with light induces oxidized purines, mainly 8-oxoGuanine, which is detected by the Fpg [39,41]. At least 2 and 3 gels were prepared for each concentration, for 2D and 3D cultures, respectively, in each experiment.

### 2.10. Statistical Analysis

Results are presented as mean with standard error of the mean (SEM) of 3 independent experiments (*n* = 3), unless otherwise mentioned. Effects were compared to nontreated cells, and statistical analysis by one-way ANOVA, multiple comparisons and post-test Dunnett were performed in GraphPad Prism 7. Comparison of 2D and 3D cultures were performed by two-way ANOVA, multiple comparisons and post-test Sidak. The *p*-values are marked by * as *p* < 0.05, ** as *p* < 0.01, *** as *p* < 0.001 and **** as *p* < 0.0001. EC_50_ values were calculated in Prism, using nonlinear regression analysis (Hill function).

## 3. Results

### 3.1. Characterization of the NPs

Characterization of the NPs was performed in water (TiO_2_-NPs 455 µg/mL, Ag- and ZnO-NPs 2.56 mg/mL), stock dispersions (TiO_2_-NPs 455 µg/mL in TMAOH and culture medium with FBS, Ag- and ZnO-NPs 2.56 mg/mL in BSA-water) and working dispersions (212 µg/mL in medium), at 0 and 24 h after preparation. A summary of the physical and chemical characteristics of the pristine NPs used is shown in Table 1.

#### 3.1.1. Electron Microscopy Analysis for Size and Shape of the NPs

**Figure 1:**
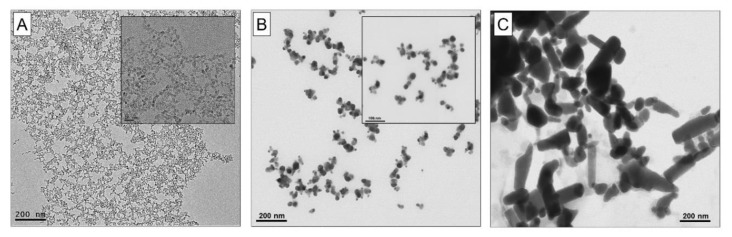
Representative transmission electron microscopy (TEM) images of (**A**) quasi-spherical TiO_2_-NPs, and representative scanning transmission electron microscopy (STEM) images of (**B**) spherical Ag-NPs and (**C**) irregular ZnO-NPs in pure water. Scale bar = 200 nm. Scale bar in inserts: (**A**) 20 nm and (**B**) 100 nm.

The primary size and shape of the NPs in water were determined by electron microscopy imaging (Figure 1). The TiO_2_-NPs were quasi-spherical, with a mean diameter of 5.54 ± 0.98 nm (Figure 1A), the Ag-NPs were spherical (Figure 1B) and the ZnO-NPs were aggregated with irregular shapes (Figure 1C).

#### 3.1.2. UV-Vis Spectroscopy for Analysis of Particle Stability

**Figure 2:**
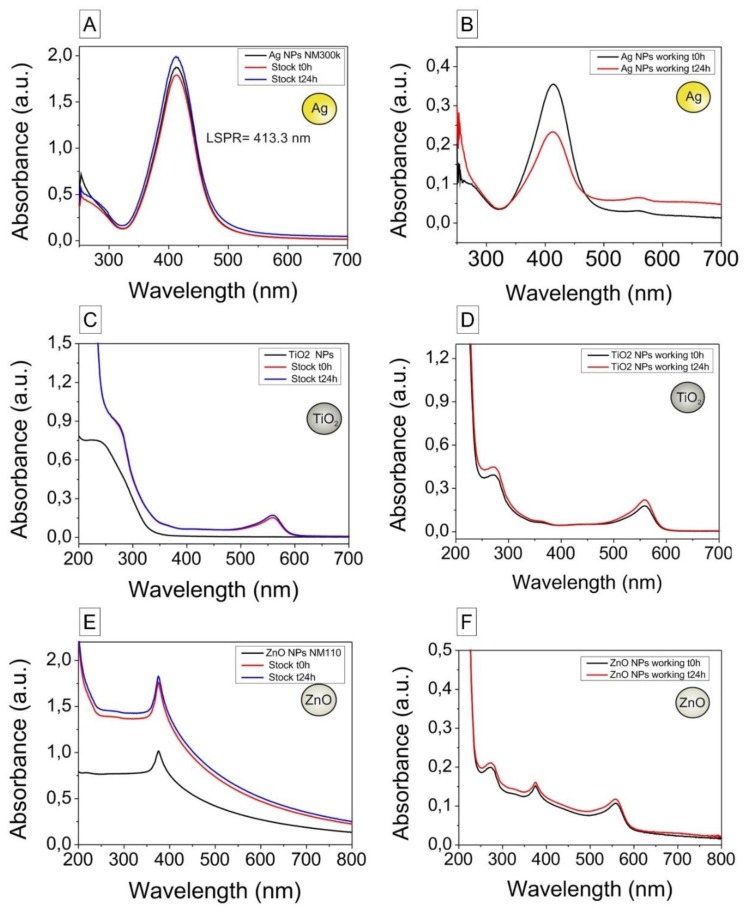
UV−vis spectra of the nanoparticle dispersions diluted in pure water (t = 0 h), as stock dispersions (t = 0 and 24 h) and as working dispersions (t = 0 and 24 h). Samples were diluted 1:200 (Ag), 1:10 (TiO_2_) and 1:20 (ZnO) in pure water for analysis. (**A**) Ag-NPs in pure water and stock dispersion (2.56 mg/mL). (**B**) Ag-NPs working dispersion (212 μg/mL, t = 0 and 24 h). (**C**) TiO_2_-NPs in water and stock dispersion (455 μg/mL, t = 0 and 24 h). (**D**) TiO_2_-NPs working dispersion (212 μg/mL, t = 0 and 24 h). (**E**) ZnO-NPs in pure water and stock dispersion (2.56 mg/mL, t = 0 and 24 h). (**F**) ZnO-NPs working dispersion (212 μg/mL, t = 0 and 24 h). The peak at 560 nm can be ascribed to the presence of phenol red in the culture medium.

UV−vis spectra of the NP dispersions prepared in pure water (t = 0 h), as stock dispersions (t = 0 and 24 h) and as working dispersions (t = 0 and 24 h) are shown in Figure 2. When comparing Ag-NPs diluted in pure water and in water with BSA (stock), no red-shift is observed (Figure 2A,B). The red-shift is indicative of the formation of a dense dielectric layer onto the NP surface consistent with the absorption of proteins on their surface, and no stable protein corona formation was thus measured for Ag-NPs, which can be ascribed to the presence of polyethylene glycol at their surface. The UV-vis spectra of Ag-NPs working dispersions have an increased absorbance signal at high wavelengths and a decrease in the peak intensity, indicative of aggregation. The UV-vis spectra of TiO_2_- and ZnO-NPs (Figure 2C–F) lack absorption peaks in the visible region, and no changes in time were observed. The small peak that appears in the visible region around 500 nm, as shown in Figure 2B,D,F, is due to the presence of phenol red in the culture medium.

#### 3.1.3. Hydrodynamic Diameter and Zeta Potential

The hydrodynamic diameter and zeta potential of the Ag-, ZnO- and TiO_2_-NPs in pure water (t = 0 h only) are summarized in Supplementary Table S3; NP stock dispersions are in Table 2, and NP working dispersions are in Table 3. Representative size distribution curves are shown in Supplementary Figures S1 and S2.

The hydrodynamic diameter (by intensity) was for all NPs higher than the pristine NP size. At the start of the experiment, the mean hydrodynamic diameter (by intensity) was 54.2 nm for Ag-NPs, 373.8 nm for ZnO-NPs and 193.6 nm for TiO_2_-NPs. The hydrodynamic diameter of the TiO_2_- and ZnO-NPs increased slightly between 0 and 24 h, for both the stock and working dispersions. In contrast, the increase in hydrodynamic diameter of the Ag-NPs was strong between 0 and 24 h, and the polydispersity index (PDI) was relatively high at 24 h, indicating a broader size distribution. The mean hydrodynamic diameter of samples without NPs showed the presence of proteins in the dispersions, measured with high variations (BSA-water 152.7 nm ± 43.0 nm with PDI 0.406 ± 0.003; medium 120.2 nm ± 59.9 nm with PDI 0.299 ± 0.128).

The zeta potential measurements also showed an evolution of the NPs’ surface charge. A drop in the surface charge, toward the average value of proteins, was observed when comparing the dispersions without proteins (Supplementary Table S3) with stock (Table 2) and working dispersions (Table 3). Zeta potential curves are shown in Supplementary Figure S3. The zeta potential of NP free BSA-water was −2.15 ± 1.01 mV, and the corresponding value of medium was −5.77 ± 2.49 mV.

#### 3.1.4. ICP-MS Analysis of Dissolved Ag and Zn in NP Dispersions

The concentrations of dissolved Ag and Zn in the <3 kDa filtrates were analyzed by ICP-MS. Medium without NPs had a Zn concentration of 25.7 µg/L (25.4–150.9 µg/L) or 0.4 µM (0.2–2.3 µM) whereas the Ag concentration was below the detection limit (<0.006 µg/L). A substantial amount of Zn was measured in the filtrate of the medium with added ZnO-NPs, ranging from 8 to 87 µM. In the filtrates from medium with added Zn-NPs (10–100 µg/cm^2^), the Zn concentration was nearly the same (79 to 87 µM) (Table 4). The concentrations of dissolved Ag in the filtrate of the medium with added Ag-NPs ranged from 0.00008 to 0.014 µM (Table 4).

### 3.2. Cytotoxicity of Ag-NPs, ZnO-NPs and TiO_2_-NPs in 2D and 3D Cultures

**Figure 3:**
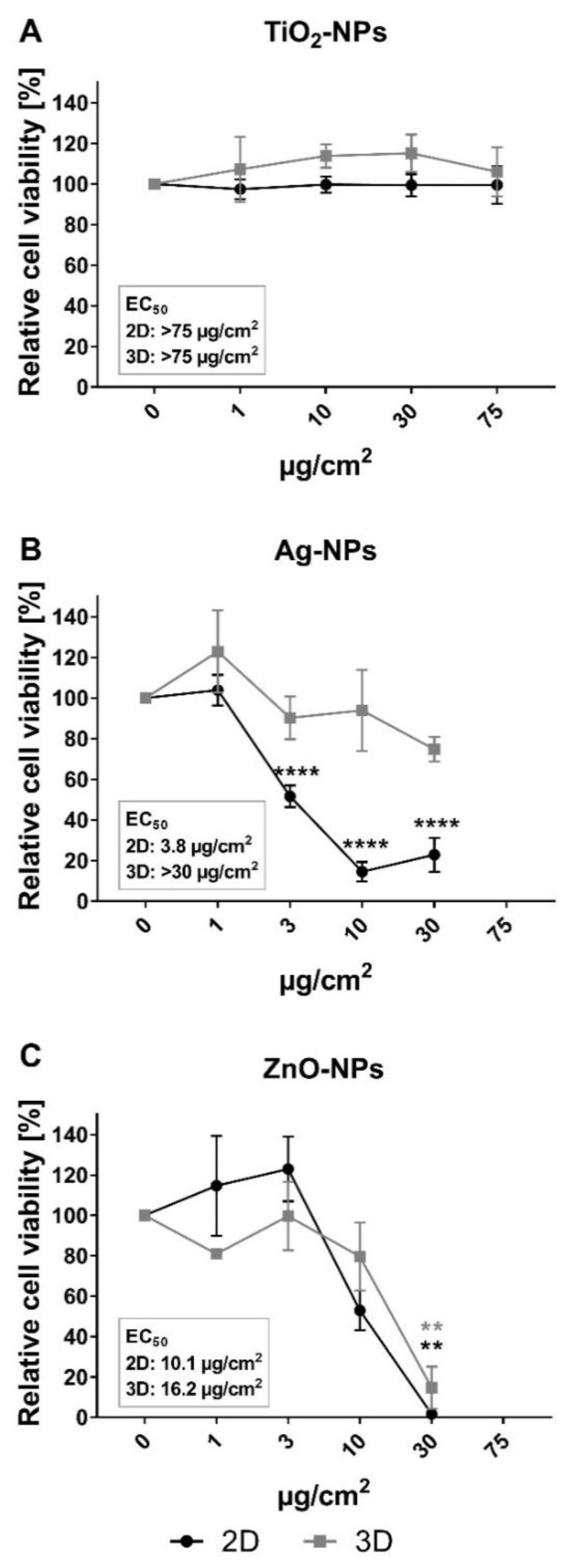
Cytotoxicity of TiO_2_-, Ag- and ZnO-NPs measured by alamarBlue assay in 2D and 3D HepG2 cultures. Cell viability was measured as metabolic capacity and calculated relative to negative control cultures (set to 100%). (**A**) No significant effects were seen on the viability of 2D (black curve) and 3D (gray curve) cultures after 24 h exposure to TiO_2_-NPs. The cell viability was reduced after 24 h incubation with (**B**) Ag-NP and (**C**) ZnO-NP for both 2D and 3D cultures. The effect of the exposure was significantly different in 2D and 3D cultures after exposure to Ag-NP at concentrations 10 and 30 µg/cm^2^, evaluated by two-way ANOVA with post-test Sidak. Values are presented as mean ± SEM of 2–6 independent experiments: (**A**) *n* = 2, 3, (**B**) *n* = 4–6 and (**C**) *n* = 3. The concentration 75 µg/cm^2^ was excluded for testing of Ag-NP and ZnO-NP (b and c) because of high cytotoxicity in previously published experiments [36]. ** *p* < 0.01; **** *p* < 0.0001.

Effects of Ag-NPs, ZnO-NPs and TiO_2_-NPs on the viability of HepG2 cells in 2D and 3D cultures were measured after 24 h exposure, using alamarBlue assay and confocal imaging. No interference of NPs with the alamarBlue assay was found (results not shown). The relative cell viability decreased in a concentration-dependent manner after exposure to ZnO- and Ag-NPs, but not for TiO_2_-NPs, in both 2D and 3D cultures (Figure 3). For ZnO-NPs, calculated EC_50_ values were in the same range for 2D and 3D cultures: 10.1 and 16.2 µg/cm^2^, respectively (Table 5). The induced cytotoxicity of Ag-NP was higher in 2D cultures compared to 3D cultures, with EC_50_ values of 3.8 and >30 µg/cm^2^, respectively (Table 5).

**Figure 4:**
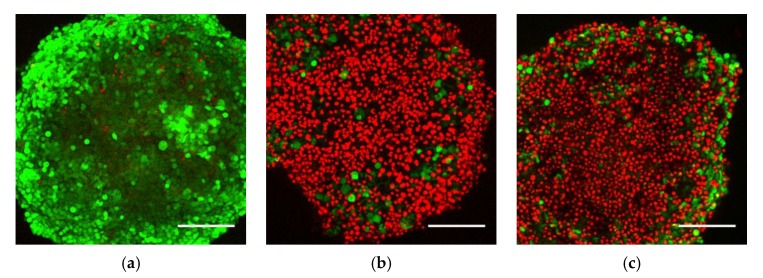
Representative confocal images of HepG2 spheroids exposed for 24 h to (**a**) culture medium, (**b**) Ag-NPs and (**c**) ZnO-NPs. Spheroids were exposed to 30 µg/cm^2^ (85 µg/mL) of Ag- and ZnO-NPs for 24 h, before staining. Dead cells were stained with propidium iodide (PI) (**red**) and viable cells with fluorescein diacetate (FDA) (**green**). The images are z-stack projections from the spheroid surface and approximately 150 µm down toward the core. An increase in number of dead cells on the surface of the spheroids was seen after exposure to Ag-NPs and ZnO-NPs. Scale bar = 200 µm. Representative images from two independent experiments (*n* = 2), each with at least three parallel spheroids.

To investigate the distribution of viable and dead cells in the spheroid culture after exposure to Ag- and ZnO-NPs, confocal microscopy and imaging was performed on exposed spheroids with live and dead cell staining by FDA and PI, respectively. Increased numbers of dead cells on the spheroid surface were seen after exposure to Ag- and ZnO-NPs at the highest concentration, and correspondingly, fewer viable cells were detected. Limited fluorescence could be detected from the spheroid core, and the viability of cells in this region could therefore not be determined. Representative images show a projection of z-stack images from the spheroid surface to approximately 150 µm into the spheroid (Figure 4). The confocal microscopy analysis showed a clear induction of cell death on the spheroid surface after exposure of Ag- and ZnO-NPs.

### 3.3. Cytotoxicity of Zn^2+^ and Ag^+^ Ion Solutions in 2D and 3D Cultures

To compare the cytotoxicity of Ag- and ZnO-NPs with corresponding salts, the alamarBlue assay was performed after exposure of HepG2 cells in the 2D model to AgNO_3_ and ZnCl_2_ solutions. Some precipitation was seen upon mixing the AgNO_3_ solution into the cell culture medium, most likely due to precipitation of AgCl due to a high presence of Cl^–^ in the medium. The relative cell viability of the HepG2 cells after AgNO_3_ and ZnCl_2_ exposure decreased in a concentration-related manner (Supplementary Figure S4). The EC_50_ values were 20.1 µM for AgNO_3_ and 362.7 µM for ZnCl_2_ (Table 5), which are higher than the amounts of dissolved Ag and Zn measured in the NP dispersions (Section 3.1.4). If we used the same concentration units as the NPs, the EC_50_ values of AgNO_3_ and ZnCl_2_ would correspond to 0.8 µg/cm^2^ (2.2 µg/mL) Ag^+^ ions, and 8.4 µg/cm^2^ (23.7 µg/mL) Zn^2+^ ions, assuming the compounds were freely dissolved in the solution. The EC_50_ values after exposure to ZnO-NPs and ZnCl_2_ were similar. The EC_50_ value for Ag-NPs exposure was higher than for AgNO_3_, showing higher cytotoxicity of the salt solution than the NPs in this test system.

### 3.4. Genotoxicity in 2D and 3D Cultures Measured by the Comet Assay

**Figure 5:**
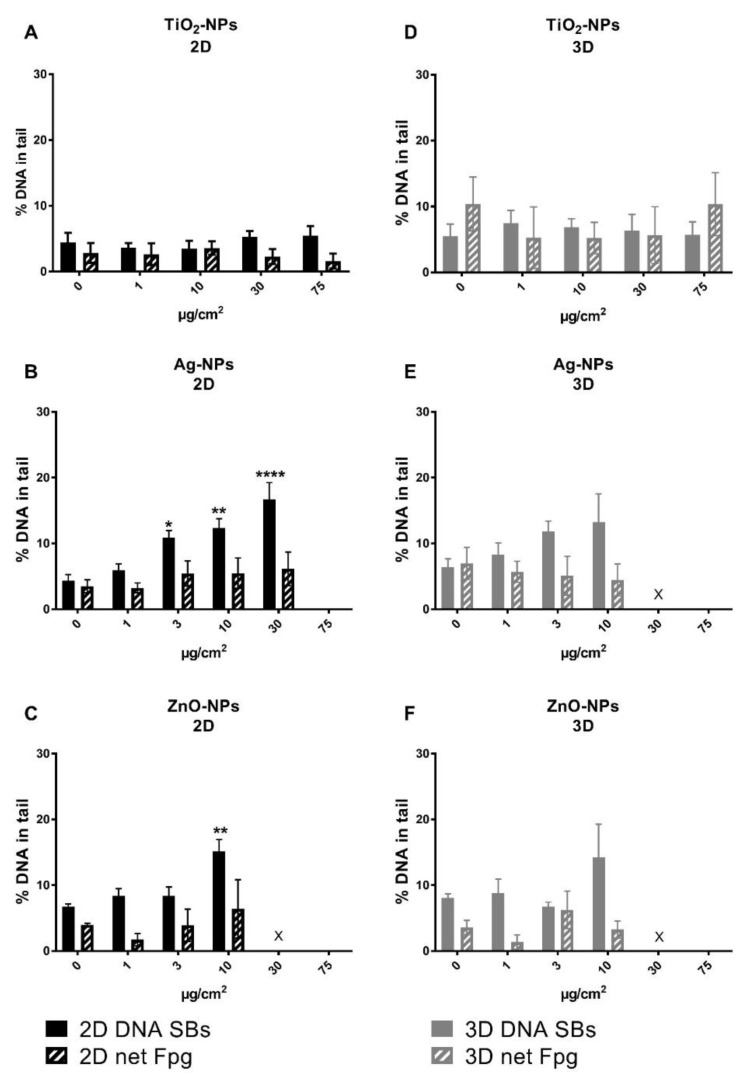
DNA damage in 2D and 3D cultures after exposure to TiO_2_-, Ag- and ZnO-NPs measured by the comet assay. The 2D (**A**–**C**) and 3D (**D**–**F**) cultures were exposed to TiO_2_-, Ag- and ZnO-NPs for 24 h. No increase in DNA damage was seen after exposure to TiO_2_-NPs. Ag- and ZnO-NPs induced an increase in DNA SBs; however, this was statistically significant only at cytotoxic concentrations. Moreover, *n* = 3 for TiO_2_- and ZnO-NPs, and *n* = 6 for Ag-NPs except at 3 µg/cm^2^, where *n* = 4. X: not measured due to cytotoxicity and too low cell number. The concentration 75 µg/cm^2^ was excluded in the experiments with Ag-NPs and ZnO-NPs (**B**,**C** and **E**,**F**) because of high cytotoxicity in previously published experiments [36]. * *p* < 0.05, ** *p* < 0.01, *** *p* < 0.001 and **** *p* < 0.0001.

The levels of DNA SBs and oxidized base lesions were measured by the enzyme-linked comet assay after 24 h exposure with NPs. In both 2D and 3D cultures, a trend with a concentration-dependent increasing level of DNA SBs was seen after exposure to Ag-NPs and ZnO-NPs; however, a statistically significant increase was found only at cytotoxic concentrations in the 2D cultures (from exposure of 3 µg/cm^2^ Ag-NPs and at 10 µg/cm^2^ ZnO-NPs in 2D cultures). No effect on the level of DNA damage was observed after exposure to TiO_2_-NPs in either 2D or 3D cultures (Figure 5). The background level of DNA damage was measured in unexposed HepG2 cells from 2D and 3D cultures and found to be similar for DNA SBs, with 5.0 ± 0.8 (2D, *n* = 9) and 6.2 ± 1.0 (3D, *n* = 7) % DNA in tail as average in all experiments (Supplementary Figure S5). For oxidized DNA base lesions, the background level was higher in 3D cultures compared to 2D, with levels of net Fpg sites at 3.7 ± 0.7 (2D, *n* = 9) and 7.6 ± 2.1 (3D, *n* = 7) % DNA in tail (Supplementary Figure S5). As a positive control for DNA SBs, cells were treated for 5 min with 50 µM H_2_O_2_; this induced a high level of DNA damage in both 2D and 3D cultures (Supplementary Figure S5). The control sample for Fpg enzyme activity, cells treated with Ro 19-8022 plus light, showed DNA damage within the expected range; the % DNA in tail was increased by at least 20 percentage points compared to without Fpg incubation (results not shown).

## 4. Discussion

There is a huge demand to develop in vitro models that more closely resemble the *in vivo* situation, for toxicity assessment of NPs and chemicals. These models should be standardized in regard to critical toxicity endpoints. Here, we have focused on the liver spheroid model and evaluated it for reliability in detecting cytotoxicity and genotoxicity of NPs.

This study investigated potential differences in induction of cell death and DNA damage, depending on whether the liver cells were cultured in 2D or 3D arrangements, by applying the enzyme-linked comet assay, accompanied with cytotoxicity tests, on HepG2 spheroids and monolayers exposed to TiO_2_-, Ag- and ZnO-NPs. HepG2 spheroids were prepared with a reproducible scaffold-free technique described in detail in Elje et al. (2019). Levels of DNA SBs in unexposed cells were found to be similar to the previous study [27]. However, 3D cultures had a higher background level of oxidized DNA lesions than 2D cultures, which can indicate a higher basal level of oxidative stress in the 3D model. This should be investigated further.

As toxicity of NPs is highly dependent upon physicochemical properties, it is important to characterize NP behavior under the given experimental conditions. The strong increase in the hydrodynamic size and the high PDI value for Ag-NPs indicate that the working dispersion of Ag-NPs had aggregated after 24 h of exposure. These results are in accordance with the UV-vis spectra, in which a decrease in intensity of the silver plasmon band, along with the increased absorbance at higher wavelengths, is shown. Ag-NP absorption is highly sensitive to the aggregation state of the NPs, due to strong surface plasmon resonance interactions between close NPs (at distances about their diameter) [44]. The UV-vis results, combined with the changes in size distribution and zeta potential, suggest that the amount of BSA protein per Ag-NP was too low to form a homogenous and dense coating, and that the stabilization of the NPs was likely to be electrostatic. Consequently, the ionic strength of the culture medium may contribute to the aggregation of the NPs. The increase in hydrodynamic diameter of TiO_2_- and ZnO-NPs after 24 h can be explained by the formation of a loosely bound soft protein corona. In terms of NP stabilization by proteins, no significant information can be drawn from the UV-vis spectra of TiO_2_- and ZnO-NPs. The wide band gap nature of these materials and their inability to absorb energy in the visible range explain the absence of absorption peaks in the visible region. Thus, the attenuation of transmitted light comes from the combination of absorption and Rayleigh scattering. Other studies using the same Ag-NPs (NM300K) and ZnO-NPs (NM110) have reported smaller hydrodynamic sizes and higher stability [6,36,43,45]. As NPs’ behavior depends on their surroundings [46], this highlights the importance of performing NP characterization with the same conditions as used in the experiments.

Exposure of HepG2 cells to TiO_2_-NPs did not induce any cytotoxicity or genotoxicity in 2D and 3D cultures. TiO_2_-NPs have also been reported in other studies to be less toxic than other nano-metal oxides [47], and their toxicity is dependent on their physicochemical properties [48,49,50]. Toxic effects have been seen in both in vitro and *in vivo* studies [36,51,52] and are not related to dissolution of metal ions [47].

ZnO-NPs exposure induced cytotoxicity to a similar extent in both 2D and 3D HepG2 cultures. Elevated DNA damage was observed in 2D and 3D cultures at the highest concentrations; however, significant induction of DNA damage was found only in 2D cultures and at cytotoxic concentrations. Similarly, exposure to Ag-NPs induced cell death and a concentration-dependent increase in DNA damage, though statistically significant only at cytotoxic concentrations. The reduced viability seen with the alamarBlue assay was strongest in 2D cultures and did not reach statistical significance in the 3D cultures. However, when the spheroids were examined with confocal microscopy, many dead cells were seen at the surface of the exposed 3D cultures. As expected for a relatively complex model that closely resembles organ structure, higher variability was found in 3D cultures compared to 2D cultures. This can explain why statistically significant results were more difficult to achieve.

Significant induction of DNA SBs after ZnO- and Ag-NPs exposure was seen only at cytotoxic concentrations, in contrast to previously reported results for A549 and TK6 cells with the same NPs and nearly identical comet assay protocols [36]. This can be explained by the cell lines used, as the HepG2 cells seem to be less sensitive to genotoxic compounds than A549 and TK6 cells. Cowie et al. studied genotoxic response to metal and polymeric NPs in human and mammalian cells of different origin and found large differences in sensitivity of cells, with TK6 cells giving one of the best concentration-dependent response [53]. The discrepancy can also be related to the differences in cell cycle and exposure times. As demonstrated in a study applying the comet assay with Fpg to spheroids of primary liver cells (InSphero model), the genotoxic effect of NPs increased after repeated or longer exposures [6]. This was shown using the same Ag- and ZnO-NPs as in the present study, in addition to TiO_2_-NPs and carbon nanotubes. Ag- and ZnO-NPs were the most potent NPs for inducing DNA SBs in the spheroidal culture, showing similar effects as in 2D cultured C3A HepG2 derivative cells [6,54]. Ag-NPs also induce an increase in DNA oxidation [6]. The presence of non-parenchymal cells can possibly explain the higher response to the NPs in the InSphero model compared to the HepG2 spheroids. The HepG2 spheroids show relatively high metabolic capacity and appear to be a good advanced in vitro model for the liver [21,22,23,24]. The commercial primary cell InSphero co-culture model is more complex than the HepG2 spheroids. However, the HepG2 spheroids used in this study are easy to prepare, have low costs and high interlaboratory reproducibility [27], and are thus a convenient and reliable alternative to commercial models.

Underlying mechanisms of NP toxicity include oxidative stress and dissolution of the NPs. In the case of metal or metal oxide NPs, toxicity can be caused by dissolution of ions, direct action of the NPs or interaction between NPs and the cellular environment [1,47,55,56,57]. Both ZnO- and Ag-NPs are generally found to induce toxicity in 2D cultures, as well as liver damage *in vivo* [17,18,55,56]. For metal NPs, there is always a question of whether toxicity is due to direct effect of the NPs or to dissolved ions. We found that a substantial number of ions was released at the start of the exposure (Table 4), with dissolved Zn concentrations of 10–100 µg/cm^2^. Increased intracellular Zn^2+^ levels resulting from dissolution of ZnO-NPs have been reported to be correlated with high levels of reactive oxygen species (ROS) and apoptosis [56]. However, Sharma et al. (2012) found that the released zinc ions were less important for the toxic effects of ZnO-NPs in HepG2 cells [58]. Other critical factors for cytotoxicity and genotoxicity of ZnO-NPs are size, shape, surface composition and semiconductor characteristics [55,56,58]. Other studies have showed that ZnO-NPs dissolve rapidly in cell culture medium DMEM, with a subsequent slow increase over time [59], and that the dissolution is dependent upon factors such as pH, ionic strength and HCO_3_^−^ and HPO_4_^2−^ concentrations, and less on the initial NP concentration [60]. That the level of dissolved Zn reached a plateau is most likely explained by a saturation of dissolved zinc in the medium. In the 2D model, ZnCl_2_ and ZnO-NPs had similar EC_50_ values for cytotoxicity by alamarBlue assay. The level of dissolved Zn from the ZnO-NPs corresponded to a nontoxic concentration of ZnCl_2_. These results indicate that the cytotoxic effect of ZnO-NP was caused by either by ZnO-NPs or the combination of ZnO-NPs and Zn^2+^ ions, and not only by released Zn^2+^ ions.

Dissolution and release of ions has been linked also with toxicity induced by Ag-NPs [47,55]. Oxidation of Ag(0) on the surface of the NPs, as well as other forms of interactions, will lead to particle corrosion and release of Ag^+^ [61,62,63,64], which, after cellular uptake, can cause mitochondrial dysfunction [64,65]. In the present study, low levels of dissolved Ag were found in the Ag-NPs exposure dispersions shortly after exposure, and the amounts were lower than the measured EC_50_ for cytotoxicity of AgNO_3_. Higher amounts of dissolved Ag have been found in other studies using the same Ag-NPs [6,37,54], and the differences may be related to distinct exposure media, different incubation times and sample preparation. However, most Ag^+^ released from the NPs will not remain freely dissolved in the cell culture medium, due to the high ionic strength of cell culture media and presence of halides (0.12 M total dissolved Cl [66]), amino acids and proteins. Unbound Ag^+^ will precipitate as AgCl and Ag_2_S [62,65] or bind to proteins due to high affinity to thiol groups (SH-groups) [67]. Precipitation was observed when preparing AgNO_3_, and a substantial part of the AgNO_3_ solution was most likely precipitated AgCl (K_SP_ 1.77 × 10^−10^ M^2^ [68]) and not freely dissolved Ag^+^ ions [69,70]. For the Ag-NPs dispersion, precipitated nano- and microcrystals may have been trapped in the filter during sample preparation for ion analysis and thus not detected as dissolved Ag. Consequently, the low level of freely dissolved Ag cannot be correlated with the persistence of the Ag-NPs in the presence of halides. Thus, it is unclear to what extent the Ag-NPs dissolve under the given experimental conditions. Oxidative stress is a likely underlying mechanism of Ag-NP-induced toxicity [64], as the corrosion of Ag-NPs is REDOX active and produces ROS [49,50]. A mechanism for induction of ROS production of Ag-NPs consists of interactions with proteins, subsequent altered protein function [71] and activation of signaling pathways involved in ROS production [64]. An increased intracellular level of ROS can activate cell-death-regulating pathways, such as p53, AKT and MAP kinase [72]. Thus, it is not clear if the toxicity of Ag-NPs in 2D and 3D cultures was caused by the ions released from the Ag-NPs, the Ag-NPs or both.

The differences in sensitivity to NP-induced toxicity on 2D and 3D cultures could possibly be related to the exposure scenarios. While the cells in the 2D cultures were growing on the bottom of flat wells, the spheroids were cultured slightly above the bottom of U-shaped wells. The cultures are most likely exposed to the same NP concentration (µg/mL) only if the exposure medium is a stable colloidal dispersion during the experimental time, which would be the case for TiO_2_- and ZnO-NPs. The Ag-NPs were aggregated at the end of the exposure time, and sedimentation of the aggregates would increase the concentration of NPs reaching the cells in the 2D cultures, while decreasing it for the 3D cultures. Possibly this can explain the stronger effect on viability of the 2D cultures compared with 3D cultures. As spheroidal cultures are exposed directly only on the spheroid surface, the exposure of cells in the interior is dependent on penetration of the compound inside the spheroid. Toxicity to cells in the interior of the spheroid could also occur via cell signaling pathways activated in the cells on the surface of the spheroid. As shown in Elje et al., short exposure to H_2_O_2_ was not sufficient to induce the same levels of DNA SBs in HepG2 2D and 3D cultures. The induced damage was around ten times higher in the 2D cultures, possibly explained by too short a time for the compound to reach the cells in the interior of the spheroid [27]. Fleddermann et al. found that SiO_2_-NPs were distributed through the whole HepG2 spheroid when the NPs were mixed with cells before spheroid formation. However, when exposing the already formed spheroids for 24 h, NPs were seen only to a depth of 20 µm [26]. Cell types, cell densities, physicochemical characteristics of the NPs (including size distribution) and ion release may influence the penetration inside the spheroid [73,74,75,76,77].

Several studies have shown differences in sensitivity to induced toxicity in 3D and 2D cultures [25,27,28,29,73,78]. We have previously found similar cytotoxicity in 2D and 3D HepG2 cultures, but higher sensitivity in the 3D culture for induced DNA damage by MMS [27]. Increased sensitivity in genotoxicity was also seen after exposure to 11 chemicals in a HepaRG spheroid model [29]. In agreement with this, benzo(a)pyrene and 2-amino-1-methyl-6-phenylimidazo(4,5-b)pyridine, which both require metabolic activation for induction of genotoxicity, induced a higher micronucleus frequency in HepG2 spheroids compared to monolayer cultures [28]. Other studies showed a greater resistance of the 3D cultures to toxicity of various drugs and chemicals [73,78]. Thus, the development of advanced 3D models for toxicity testing in vitro can give a more realistic model for human hazard and risk assessment. Slight modifications of experimental protocols may be needed for 3D cultures when comparing them to 2D cultures, to be able to control the concentration of the tested NPs or chemical that reaches the cells. Introduction of non-parenchymal cells, such as endothelial cells, stellate cells or macrophages, in co-cultures with hepatocytes, will make the model more complex and can further increase the relevance to the human liver.

## 5. Conclusions

With the increasing production of NPs and, thus, the risk of exposure to humans, the development of advanced in vitro models is especially important with respect to time, costs and the 3Rs. This study has shown that the HepG2 spheroid model can be applied successfully for the testing of NP-induced cytotoxic and genotoxic effects. The toxic responses in 2D and 3D cultures can be different, as seen after exposure to Ag-NPs where the 3D cultures were more resistant, but also similar, as TiO_2_-NPs induced no effect, and ZnO-NPs induced a strong cytotoxic effect in both models. The 2D cultures reflected concentration-dependent responses better; higher variability was seen in 3D cultures, and thus statistically significant results were more difficult to achieve. Ultimately, 3D cultures may be a more realistic model when compared to the human liver, as the spheroid model involves more complex cell arrangements and exposure scenario. The HepG2 spheroid model is thus a promising 3D model for use in nanotoxicology.

## Figures and Tables

**Table 1:** Characterization of pristine nanoparticles (NPs). TiO_2_-NPs were provided by ICN2, Ag-NPs by Fraunhofer IME and ZnO-NPs by JRC. TMAOH: tetramethylammonium hydroxide. PGT: polyoxyethylene glycerol trioleate. PEG: polyethylene glycol, TEM: transmission electron microscopy. NA: not available.

NP	Code	Product Type	Solvent	Polymorph	Morphology	Surface Functionali-zation	TEM Diameter (nm)	Surface Area (m^2^/g)	Ref.
TiO_2_	-	Dispersion	TMAOH	Anatase	Quasi-sphere	TMAOH	5.54 ± 0.98	NA	
Ag	NM300K	Dispersion	PGT (4%), Tween 20 (4%)	Metallic	Sphere	PEG	<20 nm	NA	[42]
ZnO	NM110/JRCNM01100a	Powder	-	Zincite	Variable	Uncoated	147 ± 149	12.4 ± 0.2	[43]

**Table 2:** Hydrodynamic diameter and zeta potential (ZP) of nanoparticle (NP) stock dispersions (concentrations: TiO_2_ 455 μg/mL; Ag and ZnO 2.56 mg/mL). For analysis, samples were diluted 1:10 in pure water. Numbers are given as mean ± standard deviation (SD) (*n* = 3). PDI: polydispersity index, a.u.: arbitrary unit.

NP	Time (h)	Hydrodynamic Diameter (nm), by Intensity	PDI (a.u.)	ZP (mV)
TiO_2_-NPs	0	193.6 ± 6.2	0.262 ± 0.013	−16.1 ± 1.80
	24	207.4 ± 43.1	0.242 ± 0.008	−14.3 ± 0.61
Ag-NPs (NM300K)	0	54.2 ± 3.48	0.364 ± 0.023	−9.84 ± 3.94
	24	57.5 ± 1.50	0.459 ± 0.026	−8.79 ± 2.41
ZnO-NPs (NM110)	0	373.8 ± 21.5	0.199 ± 0.046	−15.8 ± 0.70
	24	400.1 ± 11.9	0.166 ± 0.032	−14.8 ± 0.30

**Table 3:** Hydrodynamic diameter and zeta potential (ZP) of nanoparticle (NP) working dispersions (concentration 212 µg/mL, corresponding to 75 µg/cm^2^). For analysis, samples were diluted 1:10 in pure water. Numbers are given as mean ± standard deviation (SD) (*n* = 3). PDI: polydispersity index; a.u.: arbitrary unit.

NP	Time (h)	Hydrodynamic Diameter (nm), by Intensity	PDI (a.u.)	ZP (mV)
TiO_2_-NPs	0	217.3 ± 27.3	0.285 ± 0.013	−5.73 ± 1.62
	24	189.8 ± 16.2	0.235 ± 0.022	−7.49 ± 2.63
Ag-NPs (NM300K)	0	37.3 ± 0.04	0.283 ± 0.065	−14.4 ± 1.99
	24	508.8 ± 29.5	0.452 ± 0.095	−20.1 ± 1.45
ZnO-NPs (NM110)	0	346.1 ± 9.6	0.258 ± 0.020	−23.8 ± 0.30
	24	338.0± 21.7	0.281 ± 0.032	−24.9 ± 0.25

**Table 4:** Concentrations of dissolved Ag and Zn in dispersions of Ag- and ZnO-nanoparticles (NPs) in cell culture medium. Zn concentrations in medium without NPs was 25.7 µg/L (25.4–150.9 µg/L) or 0.4 µM (0.2–2.3 µM). Ag content was below the limit of detection (<0.006 µg/L). Numbers are given as median (interquartile range) (*n* = 3). * Theoretical concentration of total Ag or Zn in the dispersion (not ZnO).

NP	Nominal Concentration			Measured Dissolved Ag/Zn Concentration (<3 kDa)		
	µg/cm^2^	µg/L *	µM *	µg/L	µM	% of Nominal
Ag-NPs (NM300K)	1	2827.4	26.2	0.0090 (0.0089–0.0399)	0.00008 (0.00008–0.00037)	0.0003
	10	28274.3	262.1	0.0920 (0.0804–1.2723)	0.001 (0.001–0.012)	0.0003
	30	84823.0	786.3	1.49 (0.88–4.08)	0.014 (0.008–0.038)	0.0018
	100	282743.3	2621.1	0.20 (0.12–6.98)	0.002 (0.001–0.065)	0.00007
ZnO-NPs (NM110)	1	2271.5	34.7	519.7 (428.0–611.3)	7.9 (6.5–9.4)	22.9
	10	22715.0	347.5	5166.9 (4998.8–5693.0)	79.0 (76.5–87.1)	22.7
	30	68144.9	1042.4	5177.1 (4898.0–5436.3)	79.2 (74.9–83.2)	7.6
	100	227149.6	3474.8	5700.5 (5627.4–6057.8)	87.2 (86.1–92.7)	2.5

**Table 5:** EC_50_ values from alamarBlue assay in HepG2 2D and 3D cultures after 24 h exposure to TiO_2_-NPs, Ag-NPs or ZnO-NPs. EC_50_ values of metal compartments are given in parentheses.

Substance	2D			3D		
	EC_50_ (µg/cm^2^)	EC_50_ (µg/mL)	EC_50_ (µM)	EC_50_ (µg/cm^2^)	EC_50_ (µg/mL)	EC_50_ (µM)
TiO_2_-NP	>75.0 (>45.0)	>212.1 (>127.1)	>2655.2	>75.0 (>45.0)	>212.1 (>127.1)	>2655.2
Ag-NP	3.8	10.7	99.2	>30.0	>84.8	>786.4
ZnO-NP	10.1 (8.1)	28.5 (22.9)	350.4	16.2 (13.0)	45.7 (36.7)	561.4
AgNO_3_	1.2 (0.8)	3.4 (2.2)	20.1	-	-	-
ZnCl_2_	17.5 (8.4)	49.4 (23.7)	362.7	-	-	-

## Supplementary Materials

The following are available online at https://www.mdpi.com/2079-4991/10/3/545/s1. Figure S1: DLS size distribution (by intensity) of nanoparticle dispersions. Figure S2: DLS size distribution (by number) of nanoparticle dispersions. Figure S3: Zeta potential of nanoparticle dispersions. Figure S4: Cytotoxicity results from alamarBlue assay on HepG2 2D model after AgNO_3_ and ZnCl_2_ exposure. Figure S5: Untreated and H_2_O_2_-exposed controls in the comet assay. Table S1: Theoretical nanoparticle concentrations applied to the 2D and 3D cultures during 24 h exposure. Table S2: Theoretical Ag and Zn content in the applied nanoparticle dispersions of Ag (NM300k) and ZnO (NM110). Table S3: Characterization of NPs in pure water.
