# Supplementary figures and images for: Hepato(Geno)Toxicity Assessment of Nanoparticles in a HepG2 Liver Spheroid Model
Source: Nanomaterials (Basel). 2020 Mar 18;10(3):545. doi: 10.3390/nano10030545 (PMC7153628; doi:10.3390/nano10030545)

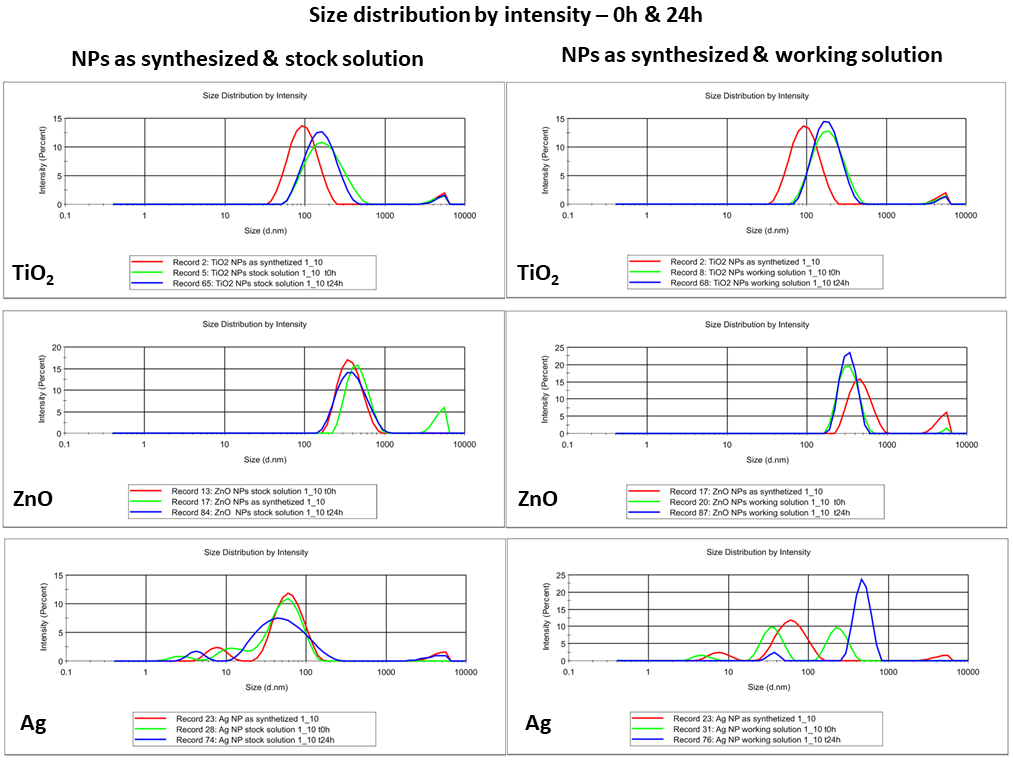

Supplement: Supplementary file 1 [file nanomaterials-10-00545-s001.zip › Fig S-1.PNG]

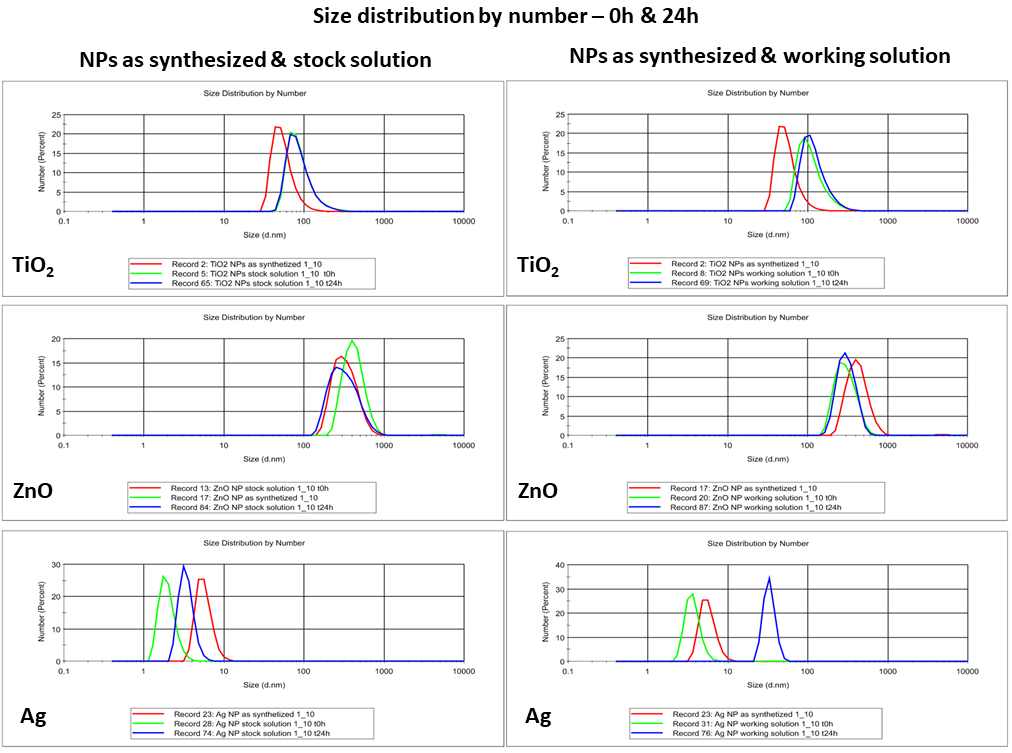

Supplement: Supplementary file 1 [file nanomaterials-10-00545-s001.zip › Fig S-2.PNG]

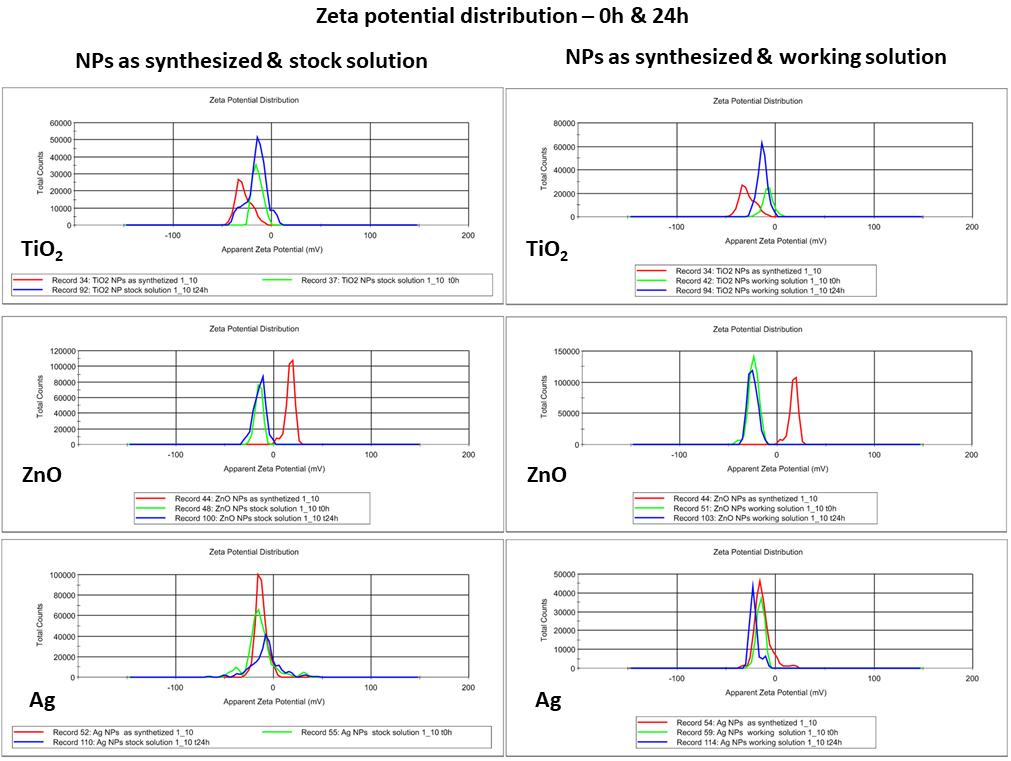

Supplement: Supplementary file 1 [file nanomaterials-10-00545-s001.zip › Fig S-3.PNG]

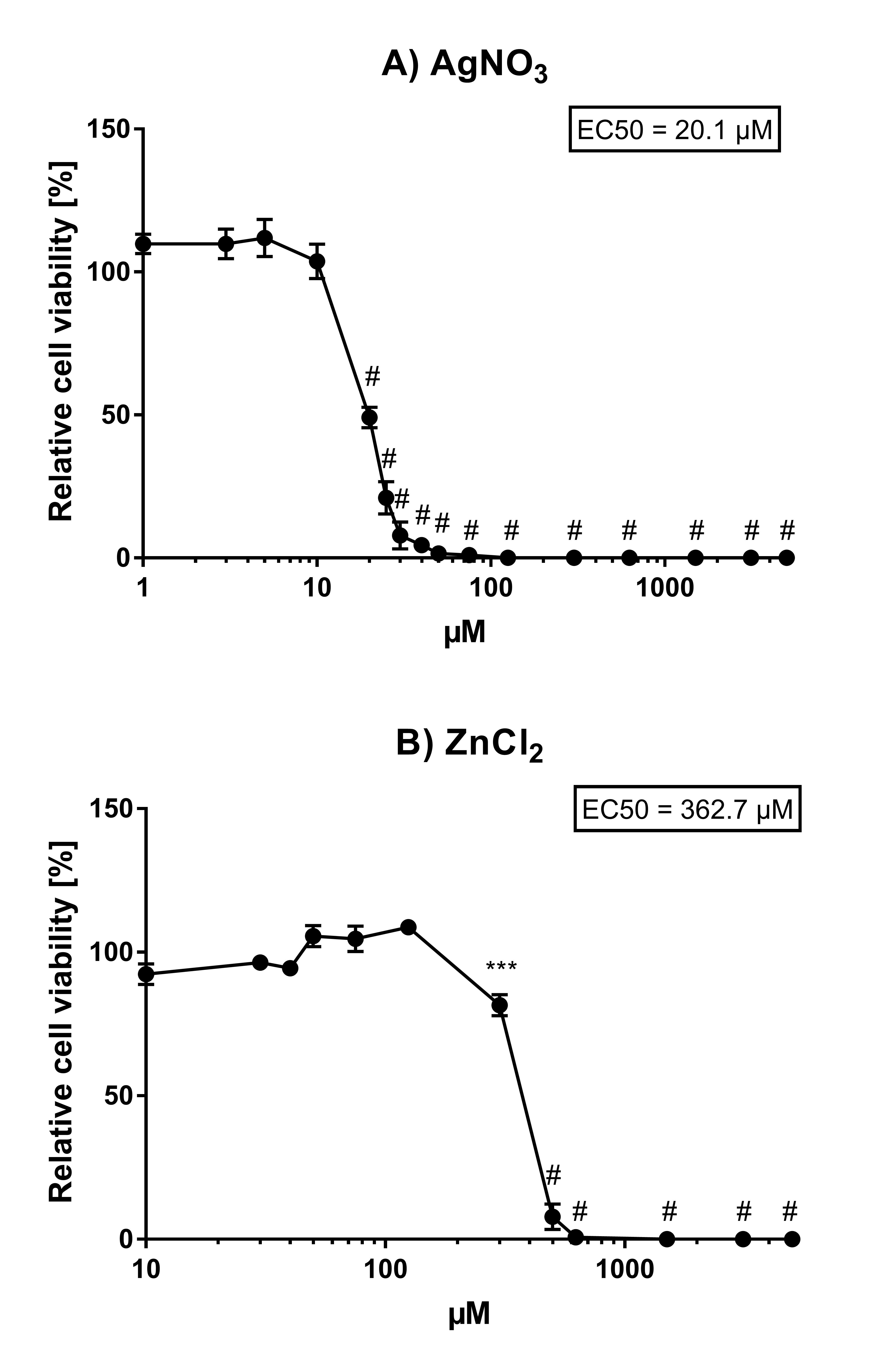

Supplement: Supplementary file 1 [file nanomaterials-10-00545-s001.zip › Fig S-4.jpg]

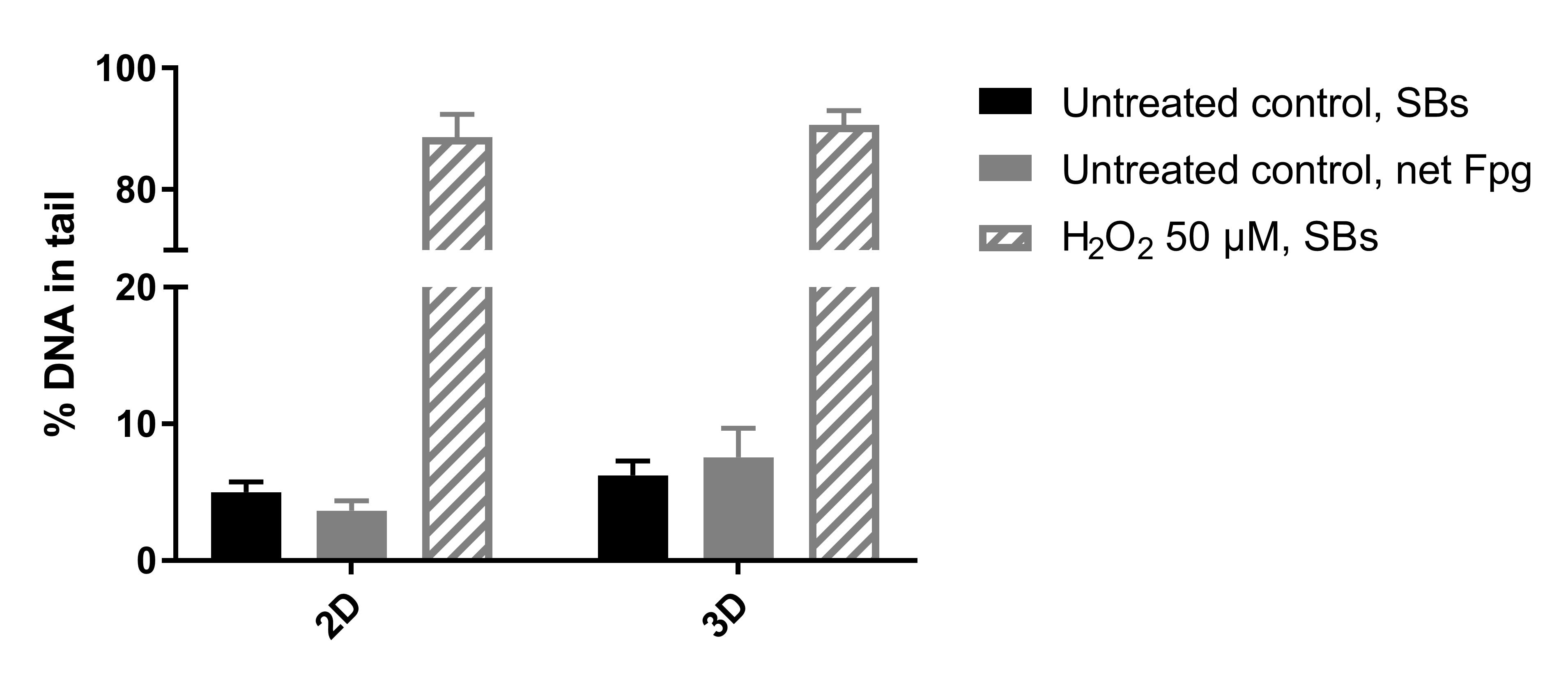

Supplement: Supplementary file 1 [file nanomaterials-10-00545-s001.zip › Fig S-5.jpg]
